# Traceability, reproducibility and clinical evaluation of Sansure Realtime HCV RNA assay

**DOI:** 10.1186/s12879-016-1390-9

**Published:** 2016-02-01

**Authors:** Xiangbo Huang, Zhongping Deng, Lu Long, Jinjun Chen, Deming Tan, Liyan Zhu, Xueying Fan, Tao Shen, Fengmin Lu

**Affiliations:** 1 Department of Microbiology and Infectious Disease Center, School of Basic Medical Sciences, Peking University, 38 Xueyuan Road, Haidian District, Beijing, 100191 China; 2 Academy for Advanced Interdisciplinary Studies, Peking University, Beijing, 100871 China; 3 Department of Infectious Diseases, Nanfang Hospital of Southern Medical University, Guangzhou, 510515 China; 4 Department of Infectious Diseases, Xiangya Hospital of Central South University, Changsha, 410008 China

**Keywords:** Hepatitis C virus, RNA, Traceability, Reproducibility, Correlation

## Abstract

**Background:**

Accurate quantitative detection of hepatitis C virus (HCV) RNA is critical for diagnosis of acute or chronic HCV infection, and for follow-up of virologic response during HCV targeted therapy. In the present study, traceability and reproducibility of a novel China-certified domestic Sansure HCV RNA diagnostic assay (Sansure, Changsha, Hunan, China) was evaluated and the clinical performance of this assay was also analyzed.

**Methods:**

Traceability of the Sansure HCV RNA assay to the WHO international standard for HCV (genotype 1a) was detected across multiple centers. Reproducibility, accuracy (the differences of observed average concentrations and expected concentrations) and precision were assessed using series dilutions of World HCV RNA performance panel WWHV303-02 (HCV-1b), WWHV303-04(HCV-2a), WWHV303-11(HCV-3a) and WWHV303-19 (HCV-6a). In addition, both Sansure HCV RNA and CAP/CTM HCV (Roche, Branchburg, NJ, USA) assays were used to detect HCV RNA in 346 EDTA anti-coagulated plasma samples from previous HCV-infected patients, during and after antiviral therapy.

**Results:**

The Sansure assay showed good traceability by agreeing with the HCV-1a WHO standard across all five concentrations tested (25, 50, 100, 1000, 10000 IU/ml). The differences between observed average concentrations and expected concentrations were all within 0.2 log_10_ IU/ml. HCV WWHV303 standards across 4 HCV genotypes (1b, 2a, 3a and 6a) were used for evaluation of reproducibility and the accuracy of the test were all within 0.2 log_10_ IU/ml. The inter-assay variations across the above 4 HCV genotypes were all less than 0.03 on each evaluated concentration, indicating good precision of Sansure HCV RNA assay. In clinical practice, concordant results were determined in 99.42 % (344/346) samples (215 positive and 129 negative samples). Two specimens with negative HCV RNA results by Sansure assay were detected positive by CAP/CTM HCV test. Correlation analysis indicated a significantly positive correlation in detected HCV RNA concentrations (*r* = 0.9439, *P* < 0.0001). HCV RNA levels in 95.35 % (205/215) specimens were within mean difference ± 1.96 SD as tested by both assays.

**Conclusions:**

With the advantages of traceability, reproducibility and lower price, Sansure HCV RNA assay represented an alternative option for HCV RNA detection in hospital and medical institution in China.

## Background

Chronic hepatitis C (CHC) and its associated cirrhosis and hepatocellular carcinoma (HCC) are major challenges of global public health [1, 2]. According to a national survey carried out in 2006, the prevalence of anti-HCV was estimated as 0.43 % in general population in mainland China [3]. However, considering a much higher prevalence of anti-HCV in high-risk groups, such as paid blood donors, patients on hemodialysis, patients with hemophilia, injection drug users (IDUs), persons with multiple sex partners and men who have sex with men and HIV-infected subjects, the “true” prevalence of anti-HCV was re-estimated to be around 1 % in China [4]. Thus, the absolute anti-HCV positive population may reach approximately 13 million at present. The common HCV genotype in Chinese CHC patients was genotype 1b, accounting for nearly half of HCV infected subjects, followed by genotypes 2a, 3a and 6a [5].

In recent years, the emergence of direct-acting antivirals (DAAs) has substantially improved the rate of SVR (sustained viral response) and thus has brought profound influence on the landscape of HCV therapy [6–10]. Therefore, it is essential to quantify HCV RNA using reliable and sensitive assays to monitor the outcomes of anti-HCV treatment, either by traditional Peg-IFNα/ribavirion (RBV) dual scheme or all-oral Peg-IFNα-free and RBV-free therapy achieved by once-daily single-tablet DAAs regimens. In addition, identification of acute HCV infection also depends on sensitive and reproducible HCV RNA detection, especially for those with low HCV viral load [11–13]. European association for the study of the liver (EASL) recommendations on treatment of hepatitis C 2014 specifically pointed out that the diagnosis of acute and chronic HCV infection was based on the detection of HCV RNA by a sensitive molecular method with detection limit lower than 15 IU/ml, and suggested that the endpoint for therapy should be determined by higher quality detection kits with lower limit less than 15 IU/ml [14].

The world Health Organization (WHO) expert committee has established an international standard for worldwide HCV RNA (genotype 1a) nucleic acid test. The standard allows results of quantitative HCV RNA detection results reporting in IU/ml by facilitating assays to be compared to an international standard. In China, in addition to the international HCV RNA quantification kits, such as Cobas Ampliprep/Cobas Taqman (CAP/CTM) HCV test kit (Roche) and Abbott Realtime HCV kit (Abbott), several domestic certified assays have also been used in clinical practice, especially in primary medical care and hospitals in un-developed regions. However, the comparability of domestic-certified HCV RNA assays to international standards is unknown. In this study, traceability and reproducibility of a novel Sansure HCV RNA quantitative fluorescence diagnostic assay (PCR-Fluorescence Probing) (Sansure) were evaluated across 4 main HCV genotypes in China. Finally, clinical performance of Sansure HCV assay was evaluated and compared with CAP/CTM HCV test (version 2) using plasma samples from chronic HCV-infected patients, during and after antiviral therapy.

## Methods

### Sansure HCV RNA assay and Roche CAP/CTM HCV test

Nucleic acid extraction, PCR reaction preparation and amplification of Sansure HCV RNA detection assay were performed on Natch S workstation (Natch S system, Sansure Biotech, Inc, Changsha, Hunan, P.R China) using protocols, reagents and software provided by the manufacturer. 0.2 ml serum/plasma samples were processed automatically. In the Sansure HCV assay, magnetic bead technology was used to extract HCV-RNA from human serum or plasma. Real-time fluorescence quantitative PCR technology was applied to quantify HCV RNA, by using a pair of specific primers, which were designed to target a conserved sequence of HCV-RNA, and a specific fluorescence probe, accompanied with other ingredients in PCR-Master mix. A total of 96 samples (16 samples/batch × 6 batches), including 90 test samples, quantitative references A-D, positive control and negative control were tested in one run. The quant references A-D were composed of inactivated positive HCV samples with different viral loads to generate ladder as reference. PCR inhibitors, including hemoglobin, bilirubin and triglyceride, have been added into samples during amplification. Testing results indicated that inhibitors had no effect on PCR amplification within the following concentration: hemoglobin (≤2 g/dl), bilirubin (≤28 mg/dl), triglyceride (≤3000 mg/dl), total IgG (≤40 g/l). In addition, positive control (Internal Control), which was designed to monitor the effect of inhibitors on PCR amplification, was added into the extraction and amplification system. Amplification results of diluted samples proved normal Ct value and no delay of internal control, confirming that all inhibitors were within the acceptable range and had no interference in the PCR system. The Natch S workstation extracted nucleic acids and assembled PCR reactions in 130 min. The SLAN-96 instrument performed PCR, analyzed the fluorescence data, and calculated the results in 2 h. The linear range of Sansure HCV assay is ranged from 50 IU/ml to 1.0E + 08 IU/ml and its limit of detection (LOD) is set as 25 IU/ml according to manufacturer’ instruction.

The performance of the 2nd Cobas Ampliprep/Cobas Taqman (CAP/CTM) HCV Test system (Roche, Branchburg, NJ, USA) was strictly followed the instructions provided by the manufacturer, as described previously [15]. The LOD of CAP/CTM assay is 15 IU/ml and the log difference of LOD between Sansure and CAP/CTM assays is 0.22 log10 IU/ml.

### HCV RNA Standards

The 4th WHO international standard for HCV for nucleic acid amplification technology (NAT)-based assays (Code 06/102) (genotype 1a) was purchased from the National Institute for Biological Standards and Control (NIBSC) [16], which was positive for anti-HCV (detected by both Abbott Architect anti-HCV assay and Ortho HCV version 3.0 ELISA test) and HCV RNA (original concentration: 260000 IU/ml) and was used to assess traceability of Sansure HCV RNA quantitative assay. World HCV RNA performance panel WWHV303 [17] (SeraCare Life Sciences, USA) was used for evaluating reproducibility of Sansure HCV RNA quantitative assay.

### Traceability assessment

In order to determine traceability of Sansure HCV RNA assay to the WHO international standard, a panel of HCV RNA standards consisting of five dilutions 25, 50, 100, 1,000 and 10,000 IU/ml was created. The dilution was performed using HCV-negative human EDTA plasma (both HCV RNA- negative and anti-HCV negative) and tested at three different centers, including Infectious Disease Center of Peking University Health Science Center, Nanfang Hospital of Southern Medical University, and Xiangya Hospital of Central South University. The negative plasmas were tested before use and were proved without interference on PCR results. For each assay, three single run per day was performed for 3 consecutive days at each center. The Sansure kits with three different lots were used in the three study centers.

### Reproducibility assessment

The original HCV RNA concentrations of 4 panels including WWHV303-02 (HCV-1b), WWHV303-04(HCV-2a), WWHV303-11(HCV-3a) and WWHV303-19 (HCV-6a) were detected as 6.3 × 10^6^, 1.3 × 10^5^, 3.7 × 10^4^ and 2.9 × 10^5^ IU/ml by Roche 2nd CAP/CTM HCV test, respectively. The information of HCV genotypes and diluted HCV RNA concentration of World HCV RNA performance panels used in this study were listed in Table 1. Briefly, HCV-1b plasma (WWHV303-02) was diluted to 6 dilutions 25, 50, 100, 1,000, 10,000 and 100,000 IU/ml, and HCV-2a (WWHV303-04), HCV-3a (WWHV303-11) and HCV-6a (WWHV303-19) were diluted to 4 dilutions 25, 50, 100 and 1,000 IU/ml. Reproducibility evaluation of Sansure HCV RNA detection was performed by testing each panel in triplicate across the three study centers mentioned above. The Sansure kits with three different lots were used in the three study centers.

**Table 1:** Characteristics of 4 World HCV RNA Performance panels used in this study

Panel	Geno type	Original HCV RNA concentration by Cobas Ampliprep/Cobas Taqman HCV test (IU/ml)	Dilutions of panel member (IU/ml)
WWHV303-02	1b	6.3*10^6^	100000,10000,1000,100, 50, 25
WWHV303-04	2a	1.3*10^5^	1000, 100, 50, 25
WWHV303-11	3a	3.7*10^4^	1000, 100, 50, 25
WWHV303-19	6a	2.9*10^5^	1000, 100, 50, 25

### Comparison between Sansure HCV RNA assay and Roche CAP/CTM HCV test in clinical samples

The consistency between Sansure HCV RNA quantitative assay and Roche CAP/CTM HCV test was compared using 346 EDTA plasma samples from patients at least 18 years of age diagnosed as chronic HCV infection previously. The samples were collected during and after antiviral therapy with Peg-IFNα/ribavirion (RBV) dual regimen. All specimens chosen for this study were positive for anti-HCV response. HCV RNA quantitative detection by CAP/CTM HCV test was performed at Nanfang hospital and Xiangya Hospital. Sansure HCV RNA assay was performed at Infectious Disease Center of Peking University Health Science Center. Of the 346 specimens, HCV viral load was detectable in 217 samples by CAP/CTM HCV test, ranging from 1.53E + 01 IU/ml to 7.75E + 07 IU/ml, and was undetectable in 129 samples according to CAP/CTM test. Of 217 HCV RNA positive samples, 147 were detected as genotype 1b, 30 were 2a, 20 were 3a and 20 were 6a.

### Statistical Analysis

HCV RNA quantitative results were shown as log_10_ IU/ml. Means, standard deviations, the absolute difference between observed mean values and the expected concentrations and coefficients of variation were calculated by descriptive statistics. Correlation between Sansure HCV RNA assay and Roche CAP/CTM test was determined by linear regression analysis. In both traceability and accuracy analysis, observed concentrations within ±0.3 log_10_ of the excepted concentration in IU/ml were considered acceptable [18]. The precision of log_10_-transformed valid test results (within the range of the assay) was estimated at each expected log_10_ HCV RNA concentration for each genotype. The lognormal CV (%) was calculated by linear mixed effect model with site and day/run, and within-run as random effects. All statistical analyses were performed using GraphPad Prism 5.0 software (San Diego, CA, USA).

### Ethics review

The study was approved by the institutional review authorities of Peking University Health Science Center (Approval ID: PKUPHLL20090011). Informed consent was obtained from each patient. Testing was performed according to the recommendations from the Clinical Laboratory Standards Institute (CLSI) guidelines.

## Results

### Assessment of traceability

Traceability of the Sansure HCV RNA assay was compared with the the 4th HCV WHO international standard (genotype 1a). As shown in Table 2, the detection results of Sansure assay were consistent within all five diluted concentrations (25, 50, 100, 1000, 10000 IU/ml) of HCV WHO standard and the differences between observed average concentrations and expected concentrations of all dilutions were within 0.2 log_10_ IU/ml (concentration range, -0.055 to +0.198 IU/ml). Standard deviation of the Sansure HCV RNA assay ranged from 0.032 to 0.143 log_10_ IU/ml in a total of 27 repeated detections with HCV-1a RNA concentrations ranging from 25 to 10000 IU/ml, indicating good precision of the assay (Table 2).

**Table 2:** Traceability of Sansure HCV assay assessed by mean log_10_ HCV RNA viral load using the WHO standard (HCV Genotype 1a) - within assay ranges^a^

Expected HCV RNA (IU/ml)	Expected HCV RNA (log_10_ IU/ml)	Observed Mean HCV RNA (log_10_ IU/ml)	Difference of Observed and Expected HCV RNA (log_10_ IU/ml)	Standard Deviation of the mean HCV RNA (log_10_ IU/ml)
25	1.398	1.596	0.198	0.032
50	1.699	1.812	0.113	0.081
100	2	2.173	0.173	0.050
1000	3	2.969	−0.031	0.043
10000	4	3.945	−0.055	0.143

Each test had 27 results with panel members within the range of the assay

^a^ The limit of detection (LOD) of Sansure Realtime HCV assay is 25 IU/ml and its linear range is defined as from 5.0E + 01 IU/ml to 1.0E + 08 IU/ml

### Assessment of reproducibility, accuracy and precision

Four World HCV RNA Performance Panels were used for evaluation of the reproducibility of Sansure HCV RNA assay. As shown in Table 3, the observed results of Sansure assay were in agreement with the HCV WWHV303 standards across all four HCV genotypes (1b, 2a, 3a and 6a) and the accuracy (the differences between observed average concentrations and expected concentrations) of all tests was within 0.2 log_10_ IU/ml. The biggest difference across the results was -0.114 log_10_ IU/ml, which occurred in detection of WWHV303-04 (HCV-2a) on the highest concentration of 1000 IU/ml of expected HCV RNA value. In addition, among different concentrations in each genotype, the inter-assay variation of Sansure assay was always less than 0.03 (3 %), indicating that Sansure assay has a good precision across four HCV genotypes. Considering genotype 1b is the most popular HCV subtype prevailing in China and the original concentration of WWHV303-02 (HCV-1b) is up to 6.3 × 10^6^ IU/ml, standards for HCV-1b genotype was diluted to 6 dilutions (25, 50, 100, 1,000, 10,000 and 100,000 IU/ml), while the maximum dilution of other 3 genotypes (HCV-2a, 3a, and 6a) was 1000 IU/ml due to the original concentrations (Table 1). For evaluation of HCV-1b, low lognormal coefficient of variation (CV%) was found in 10000 IU/ml (1.41) and 100000 IU/ml (0.989) dilutions while high CV% was observed in 25 IU/ml (10.01), 50 IU/ml (4.023), 100 IU/ml (4.486) and 1000 IU/ml (5.055) dilutions. These results indicated that better stability of Sansure assay presented in higher viral load than in lower ones.

**Table 3:** Total precision (variance, standard deviation and CV %) of PCR assays for HCV genotypes 2b, 1a, 3a and 6a (mean log10 HCV RNA viral load) at different key concentrations - within assay ranges

HCV genotype	Expected HCV RNA (IU/ml)	Expected HCV RNA (log10 IU/ml)	No. tests performed	Observed mean HCV RNA (log10 IU/ml)	Difference of observed and expected HCV RNA (log10 IU/ml)	Total precision variance (SD) of log10 HCV RNA	Lognormal CV%
WWHV303-02 (1b)	25	1.398	9	1.372	−0.026	0.019 (0.137)	10.01
	50	1.699	9	1.665	−0.034	0.004 (0.067)	4.023
	100	2	9	1.951	−0.049	0.008 (0.088)	4.486
	1000	3	9	2.959	−0.041	0.022 (0.150)	5.055
	10000	4	9	4.015	0.015	0.003 (0.057)	1.410
	100000	5	9	5.049	0.049	0.002 (0.050)	0.989
WWHV303-04 (2a)	25	1.398	9	1.411	0.013	0.012 (0.109)	7.755
	50	1.699	9	1.710	0.011	0.009 (0.093)	5.453
	100	2	9	2.022	0.022	0.016 (0.126)	6.231
	1000	3	9	2.886	−0.114	0.017 (0.130)	4.513
WWHV303-11 (3a)	25	1.398	9	1.379	−0.019	0.013 (0.113)	8.181
	50	1.699	9	1.737	0.038	0.007 (0.080)	4.664
	100	2	9	2.044	0.044	0.019 (0.139)	6.798
	1000	3	9	2.893	−0.107	0.023 (0.150)	5.185
WWHV303-19 (6a)	25	1.398	9	1.413	0.015	0.013 (0.112)	7.958
	50	1.699	9	1.712	0.013	0.008 (0.087)	5.100
	100	2	9	1.980	−0.02	0.026 (0.162)	8.170
	1000	3	9	2.915	−0.085	0.017 (0.130)	4.478

Abbreviations: *SD* standard deviation

### Quantification of HCV RNA in clinical practice

In order to determine the clinical evaluation of Sansure assay, a total of 346 plasma samples from patients with chronic HCV infection were collected during and after antiviral therapy. Comparison of HCV RNA levels measured by Sansure assay and Roche CAP/CTM test was shown in Table 4. Among the 346 clinical samples, 344 samples were consistently tested by both assays and concordant result was determined in 99.42 % of total samples. None of the 129 matched HCV-negative samples determined by CAP/CTM HCV test gave a false-positive HCV RNA result with Sansure assay, achieving a specificity of 100 %. Only two specimens with negative HCV RNA results by Sansure assay were detected positive by CAP/CTM HCV test (HCV-3a for 1.53E + 01 IU/ml and HCV-6a for 1.82E + 01 IU/ml respectively), which indicated that Sansure assay was slightly less sensitive than CAP/CTM test, especially in low viral loads samples.

**Table 4:** Clinical performance of the Sansure HCV RNA assay and CAP/CTM HCV test in 346 anti-HCV positive samples

Roche COBAS HCV RNA	Sansure realtime HCV RNA	
	+	−
+	215	2
−	0	129
Total	215	131

**Fig. 1:**
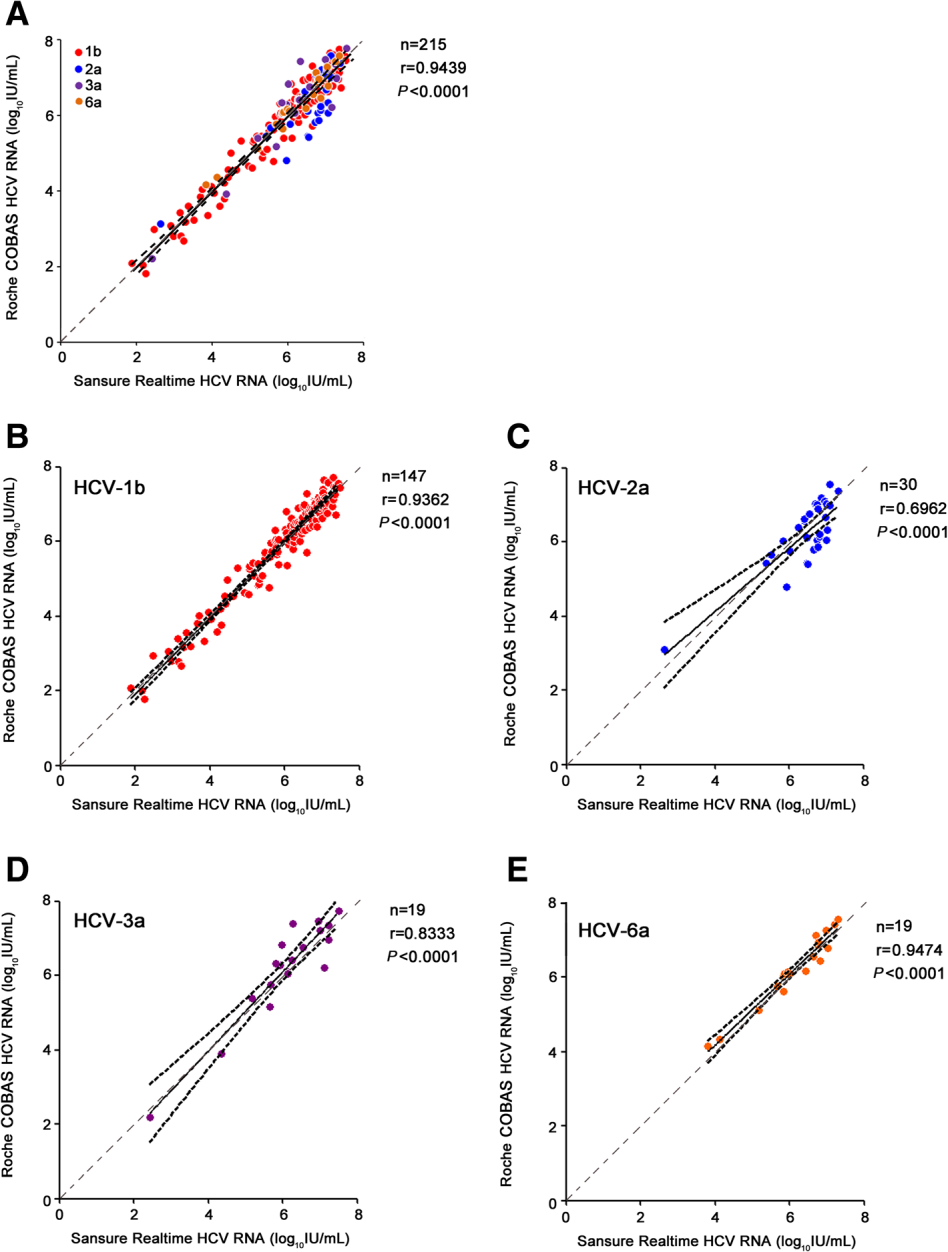
**a** High correlations were observed in measurements of HCV RNA positive samples between Sansure HCV RNA assay and Roche CAP/CTM HCV assay. Correlations between two assays from HCV RNA positive samples were further analyzed for HCV genotype 1b (**b**), 2a (**c**), 3a (**d**), and 6a (**e**) samples. 95 % confidence band (black dashed) of the best-fit line (black solid line) was shown. Quality line was indicated by grey dashed line

**Fig. 2:**
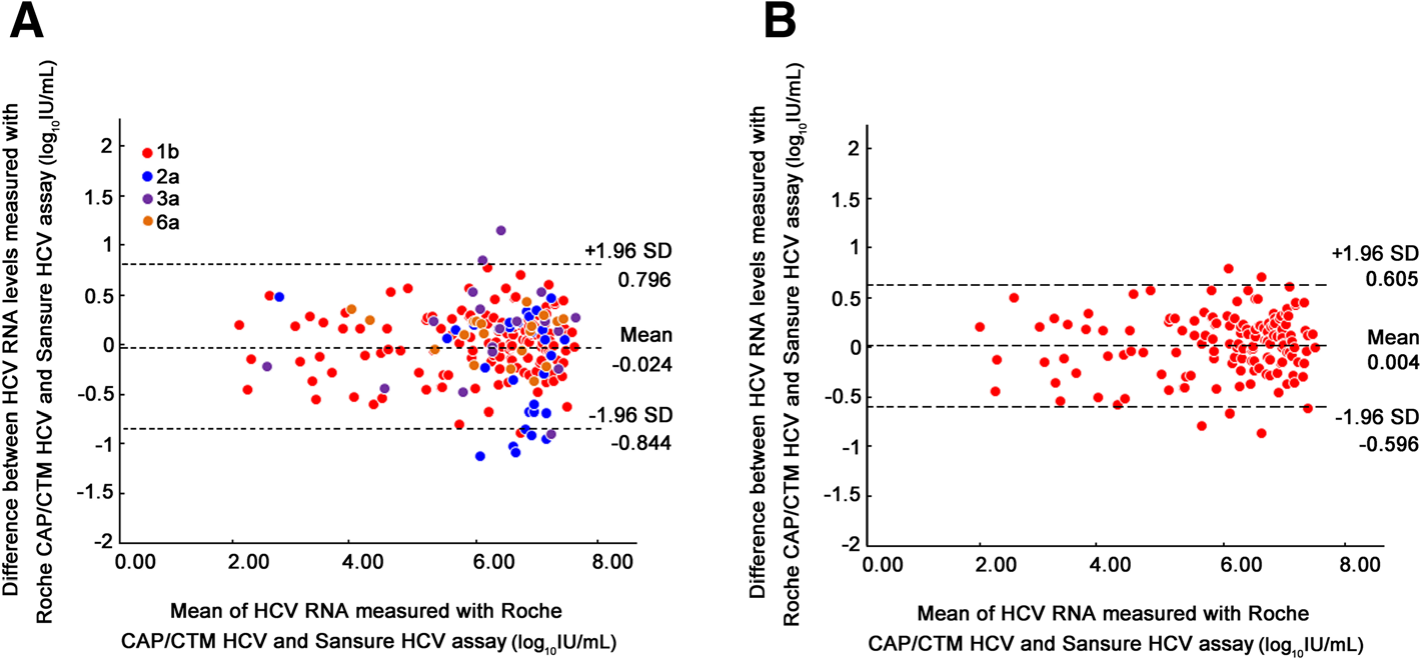
Bland-Altman analysis of hepatitis C virus (HCV) RNA level were measured with Sansure HCV and CAP/CTM HCV assays in total 215 clinical plasma samples (**a**) and HCV-1b samples (**b**) with detectable HCV RNA. The difference between the Sansure and CAP/CTM measurements was plotted according to the mean of the two values. Area between two dashed lines corresponded to mean difference ±1.96 SD

215 samples which were HCV RNA-positive as detected by both Sansure HCV RNA assay and Roche CAP/CTM HCV assay were further analyzed. As shown in Fig. 1, the values of HCV RNA concentrations detected by Sansure HCV RNA assay and Roche CAP/CTM HCV assay were highly correlated (*r* = 0.9439, *P* < 0.0001; Fig. 1a). We further analyzed the corrections of two assays in different HCV genotypes. 215 HCV RNA-positive samples included 147 genotype 1b, 30 genotype 2a, 19 genotype 3a and 19 genotype 6a. The correlations analysis indicated that Sansure assay were highly correlated (*P* < 0.001 for all) with CAP/CTM test for 1b (*r* = 0.9362, Fig. 1b) and 6a (*r* = 0.9474, Fig. 1c) genotypes, and to a less degree for 3a (*r* = 0.8333, Fig. 1d) and 2a (*r* = 0.6962, Fig. 1e).

Bland-Altman analysis showed that the mean different value of the 215 paired viral loads, maximal difference, and 95 % confidence interval were -0.024, 1.35 and (−0.844, 0.796) log_10_ IU/ml, respectively (Fig. 2a). The largest differences in the HCV RNA values were located in the range of mean difference ±1.96 SD (95.35 %, 205/215) and 10 were out of the range of mean difference ±1.96 SD. Since the majority of clinical samples in this study were genotype 1b, we specially performed Bland-Altman analysis for 147 1b samples, and the results indicated that mean different value, maximal difference, and 95 % confidence interval were 0.004, 1.20 and (−0.596, 0.605) log_10_ IU/ml, respectively (Fig. 2b). Only 5 values were out of the range of mean difference ±1.96 SD. These results indicated that the values of HCV RNA detected by Sansure HCV RNA assay and Roche CAP/CTM HCV assay in genotype 1b samples were closely correlated.

## Discussion

Hepatitis C virus is one of the major causes of chronic hepatitis, liver cirrhosis and fibrosis, and hepatocellular carcinoma. HCV genotyping and viral load at the time point of diagnosis are used to determine the duration of antiviral therapy and to predict the response to antiviral treatment. HCV RNA quantification plays a critical role in diagnosis of acute HCV infection, evaluating the efficacy of antiviral therapy and prognosis. Although several commercial HCV RNA quantitative reagents were available in diagnostic market in China, some quality parameters of these reagents, such as linear range, sensitivity, and precision for low-copy samples, are of great differences. After antiviral treatment, HCV RNA of some patients with negative results as detected by some domestic assays were still detected positive by some international brand commercial assays, which indicated that the sensitivity and repeatability of domestic assays need to be improved. Due to the high sensitivity, accuracy, reproducibility and wide linear range, CAP/CTM HCV test is recognized as reference for HCV RNA reagents internationally. Specially, the 2nd CAP/CTM test demonstrated good performance and higher sensitivity with lower limit of quantification of 15 IU/ml across all HCV genotypes [15]. Unfortunately, the relatively high cost of CAP/CTM HCV test is an economic burden for low-income patients. As a result, HCV RNA detection with low cost and high-through input system is becoming more and more necessary for patients and doctors.

The novel nucleic acid extraction system Natch S can process 96 samples in one test. The extracted RNA is compatible with common real-time PCR instruments with high-sensitive PCR reagents from Sansure biotech. Inc.. Alternatively, collection tubes can be loaded directly in Natch S system instead of transferring sample from collection tube to S-tube, which will save time and decrease the risk of contamination.

In this study, good traceability was achieved by Sansure assay, as shown by consistency between observed average concentrations and expected concentrations of the standard samples, which were within the acceptable range (relative deviation < ±0.2 log IU/ml) for all diluted concentrations of the standards. As for reproducibility, different concentrations of standards on genotype 1b, 2a, 3a and 6a were tested. Generally, for the genotypes prevailing in China, the differences of observed and excepted HCV RNA standards were all below 0.2 log IU/ml and all the values of lognormal CV% were lower than 15 %, indicating that Sansure assay possessed comparable accuracy and precision as CAP/CTM HCV test. However, this study also suggested that the stability of Sansure HCV assay needs further improvement for samples with lower concentrations. When evaluating the clinical plasma samples, concordant results between Sansure assay and CAP/CTM HCV test were determined in 99.42 % of total samples and the specificity was calculated to be 100 %. In addition, a significant positive correlation on the concentrations (especially for genotype 1b) detected by these two assays was observed in our study. The only discrepancy of the results was that the concentrations of two samples were failed to be detected by the Sansure assay, whereas was apparently detectable with CAP/CTM HCV test. Considering two specimens with negative HCV RNA results by Sansure assay were detected positive by CAP/CTM HCV test, we repeated five tests for each of those two samples using Sansure assay. Results from three of five times were positive for both samples. The results indicated that the accuracy and sensitivity of Sansure assay should be further enhanced, especially for detection of lower viral loads specimens. Less input sample volume used in Sansure HCV RNA assay (200 μl for Sansure vs. 500 μl for 2nd CAP/CTM) may partially contribute to the inconsistent detections of the two low-copy samples.

In short, traceability, accuracy and precision between measurements of Sansure HCV RNA assay and CAP/CTM HCV test were comparable in evaluating the 4th WHO international HCV RNA performance panel and World HCV RNA performance panel WWHV303 in this study. However, considering the fact the lower limit of quantitation was set as 25 IU/ml by the manufacture, the reliability of Sansure HCV RNA assay need to be further verified in clinical practice.

## Conclusions

In the present study, Sansure HCV RNA assay showed good traceability and reproducibility with high consistency and correlation with CAP/CTM HCV test in clinical detection. With the advantages of superior flexibility, traceability, reproducibility and lower price, Sansure HCV RNA assay represent an alternative option for HCV RNA detection in hospital and medical institution in China.

## Abbreviations

CAP/CTM: Cobas Ampliprep/Cobas Taqman
CHC: Chronic hepatitis C
CV: Coefficient of variation
DAAs: Direct-acting antivirals
HCC: Hepatocellular carcinoma
HCV: Hepatitis C virus
NIBSC: National Institute for Biological Standards and Control
PCR: Polymerase chain reaction
RBV: Ribavirion
SVR: Sustained viral response
WHO: World Health Organization
